# Stability and flexibility of full-length human oligodendrocytic QKI6

**DOI:** 10.1186/s13104-019-4629-x

**Published:** 2019-09-23

**Authors:** Arne Raasakka, Petri Kursula

**Affiliations:** 1 Department of Biomedicine, Faculty of Medicine, University of Bergen, Bergen, Norway; 2 Faculty of Biochemistry and Molecular Medicine & Biocenter Oulu, University of Oulu, Oulu, Finland

**Keywords:** Myelin sheath, Protein stability, RNA-binding protein, Circular dichroism, SAXS

## Abstract

**Objective:**

Oligodendrocytes account for myelination in the central nervous system. During myelin compaction, key proteins are translated in the vicinity of the myelin membrane, requiring targeted mRNA transport. Quaking isoform 6 (QKI6) is a STAR domain-containing RNA transport protein, which binds a conserved motif in the 3′-UTR of certain mRNAs, affecting the translation of myelination-involved proteins. RNA binding has been earlier structurally characterized, but information about full-length QKI6 conformation is lacking. Based on known domains and structure predicitons, we expected full-length QKI6 to be flexible and carry disordered regions. Hence, we carried out biophysical and structural characterization of human QKI6.

**Results:**

We expressed and purified full-length QKI6 and characterized it using mass spectrometry, light scattering, small-angle X-ray scattering, and circular dichroism spectroscopy. QKI6 was monodisperse, folded, and mostly dimeric, being oxidation-sensitive. The C-terminal tail was intrinsically disordered, as predicted. In the absence of RNA, the RNA-binding subdomain is likely to present major flexibility. In thermal stability assays, a double sequential unfolding behaviour was observed in the presence of phosphate, which may interact with the RNA-binding domain. The results confirm the flexibility and partial disorder of QKI6, which may be functionally relevant.

## Introduction

In the central nervous system, long axonal segments undergo myelination by oligodendrocytes (OGs), forming the basis of rapid nerve impulse conduction. OGs extend their processes and wrap their plasma membrane around axons in a process driven by actin disassembly [1]. The plasma membrane stacks undergo compaction driven by factors like myelin basic protein (MBP) in the cytoplasm [2]. The translation of MBP and other proteins involved in myelination has to occur close to the membranes that undergo stacking [2, 3]. To achieve this, mRNAs coding for these proteins are trafficked along OG processes. Disruption of this trafficking results in dysmyelination, aberrant myelin formation [4–6].

The protein Quaking (QKI) manifests as several alternatively spliced cytosolic isoforms, the dominant ones being QKI5, QKI6, and QKI7 [7, 8]. The domain structure of QKI consists of a STAR (signal transduction and activation of RNA) domain, which is further divided into an N-terminal QUA1 dimerization domain, and KH (K-homology) and QUA2 domains that together are responsible for binding to specific 3′-UTR targets in mRNAs [9]. An additional 100 amino acids follow in the C terminus, capped by an isoform-specific stretch, which contains a nuclear localization signal in QKI5, absent in QKI6 and QKI7 [7, 9, 10]. QKI7 can induce apoptosis in OGs, and heterodimerization of QKI5 and QKI7 results in nuclear localization, suppressing apoptotic activity [11]. QKI6 functions in translational repression, being essential for OG myelination [7, 12, 13]. Lack of QKI in OGs disrupts the trafficking of myelination-related mRNAs [14], producing an aberrant myelin phenotype and related neurological issues, as demonstrated by the *quaking*^*viable*^ mouse model [7, 15].

Structural studies on the QUA1 domain and its *Xenopus* homologue revealed a dimeric assembly stabilized by hydrophobic interactions [16, 17], and solution NMR studies on the KH-QUA2 region demonstrated independent folding of the two domains, which lacked major contacts with one another [18]. The crystal structure of the entire STAR domain bound to a synthetic oligonucleotide was solved, uncovering the 3′-UTR binding mode and the involved binding determinants [19]. In this structure, the KH-QUA2 region was well defined, suggesting that in the absence of RNA, its flexibility might have relevance in sensing binding motifs. Modelling studies indicated that the absence of RNA increases the STAR domain dynamics [20]. Structures of other homologous proteins have revealed KH-mediated dimerization, whereby the QUA1 domain also forms dimers, but disordered linkers join QUA1 to the KH domain [21]. To date, only the STAR domain of QKI has been structurally characterized, and the remaining regions of full-length QKI remain obscure.

We set out to study the hypothesis, based on previous structural studies and predictions, that full-length QKI6 is a flexible, partially disordered molecule. Full-length human QKI6 is a homodimeric protein with independently folded subdomains and an elongated C terminus. Its thermal stability and unfolding behaviour are affected by the presence of phosphate, which could occupy the RNA-binding site.

## Main text

### Materials and methods

#### Construct preparation

A bacterial expression construct coding for human QKI6 (UniProt: Q96PU8-9, amino acids 1–319) with an N-terminal tobacco etch virus (TEV) protease digestion site [22] was generated using Gateway cloning in the pTH27 vector [23].

#### Bioinformatics

Secondary structure prediction of the QKI6 construct was performed using psipred [24, 25]. The molecular weight (35,187.8 Da) and pI (7.10) were calculated with ProtParam [26].

#### Protein expression and protein purification

QKI6 was expressed in *E. coli* BL21(DE3) using ZYM-5052 autoinduction at 37 °C for 24 h [27]. Cells were harvested by centrifugation and resuspended in washing buffer (50 mM Tris–HCl, 300 mM NaCl, 50 mM (NH_4_)_2_SO_4_, 20 mM imidazole, 0.5 mM tris(2-carboxyethyl)phosphine (TCEP), pH 8.0) supplemented with 0.1 mg/ml lysozyme and cOmplete EDTA-free protease inhibitor cocktail (Roche). Suspensions were snap-frozen in liquid N_2_ and stored at − 80 °C until purification.

The cell suspension was lysed using ultrasonication. The lysate was clarified by centrifugation, and the soluble fraction was subjected to standard Ni-nitrilotriacetic acid (NTA) chromatography. The above-mentioned washing buffer was used and supplemented with 500 mM imidazole to elute bound proteins. His tags were removed using TEV protease [22] during overnight dialysis against imidazole-free washing buffer.

The digested protein was subjected to a second Ni–NTA chromatography. The unbound fraction was gel filtrated using a Superdex 200 16/60 HiLoad (GE Healthcare) column with 20 mM Tris–HCl, 300 mM NaCl, 1% (w/v) glycerol, pH 8.0 as running buffer. The purified protein was either used fresh, or frozen in liquid N_2_ and stored at − 80 °C. 0.5 mM TCEP was included, after QKI6 was found to be oxidation-sensitive (see below).

#### Mass spectrometry

The molecular mass of QKI6 was determined using a Micromass Q-Tof 2 after desalting using liquid chromatography. 0.5 mM TCEP was added to study oxidation sensitivity. The identity of QKI6 was verified using peptide fingerprinting and a Bruker Ultra fleXtreme mass analyzer.

#### Multi-angle light scattering

Monodispersity and molecular weight of QKI6 were determined using size exclusion chromatography-multi-angle light scattering (SEC-MALS). The chromatography was performed using an Äkta Purifier (GE Healthcare) and a Superdex 200 pg increase 10/300GL (GE Healthcare) column with 20 mM Tris–HCl, 300 mM NaCl, 0.5 mM TCEP, pH 8.0 as mobile phase. A 200-µg QKI6 sample was injected into the column at 0.4 ml/min and light scattering recorded using a Wyatt miniDAWN TREOS instrument. Concentration was determined using an on-line refractometer (Shodex RI-101). Data were analyzed using ASTRA (Wyatt).

#### Synchrotron radiation circular dichroism spectroscopy

Synchrotron radiation circular dichroism (SRCD) data were collected from 0.6 mg/ml QKI6 in 10 mM Na phosphate, pH 7.0 on the UV-CD12 beamline (ANKA, Karlsruhe, Germany) [28]. A closed circular cell (Suprasil, Hellma Analytics) of 100-µm pathlength was used for spectra recorded from 180 to 280 nm at 10 °C. Baseline subtraction and unit conversion were done with CDtoolX [29].

#### Thermal stability

The thermal stability of QKI6 was determined by differential scanning fluorimetry (DSF) as described [30–32]. We screened the effect of salt and pH combined with selected additives. A temperature range of 20–90 °C was scanned with an Applied Biosciences 7500 PCR system. The melting temperature midpoints (*T*_m_) were extracted from the curves. Every condition was measured in duplicate and contained 0.1 mg/ml QKI6.

#### Small-angle X-ray scattering

Small-angle X-ray scattering (SAXS) data were collected from 2.3 to 9.0 mg/ml samples in 50 mM Tris–HCl, 300 mM NaCl, 1% glycerol, 1 mM 2-mercaptoethanol, pH 7.5. Data collection was performed on the P12 beamline, EMBL/DESY (Hamburg, Germany). Monomeric bovine serum albumin was used as a molecular weight standard (*I*_0_ = 12,981.7; 66.5 kDa). Data reduction, processing, and analysis were performed using BioXTAS RAW [33] and ATSAS [34]. Distance distribution functions were determined using GNOM [35]. Ab initio modeling was performed with GASBOR [36] and flexible loops and termini were modeled using CORAL [37]. Data processing, analysis, and modelling details are listed in Additional file 1: Table S1.

### Results and discussion

We hypothesized that full-length QKI6 is flexible and partly disordered. Hence, we carried out a low-resolution characterization of human QKI6 in solution in the absence of bound RNA.

**Fig. 1:**
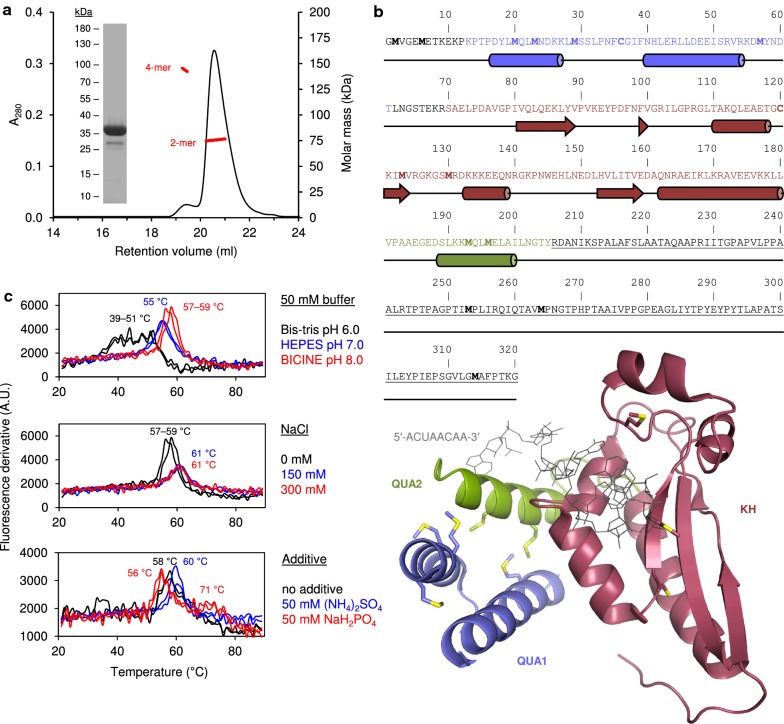
Properties of QKI6. **a** SEC-MALS profile of QKI6 displays mainly a dimer. The purity of QKI6 was determined using SDS-PAGE (inset). **b** Top: The sequence of QKI6. Secondary structure prediction is shown below the sequence. The C-terminal tail (underlined) has not been structurally characterized. Cys and Met residues are highlighted in bold. Bottom: Crystal structure of the QKI6 STAR domain in complex with an RNA oligonucleotide (PDB ID: 4jvh [19]) is presented with the subdomains annotated. Coloring matches the protein sequence. The RNA oligonucleotide (5′-ACUAACAA-3′) is shown as gray sticks. Cys and Met residues are shown as sticks. **c** Thermal stability experiments of QKI6 reveal that its *T*_m_ is increased in elevated pH and moderate salt content. Additionally, phosphate produces another melting event at ~ 71 °C. Each condition was measured twice. All pH values were tested in the absence of NaCl. All salt concentrations were tested in 50 mM BICINE, pH 8.0. The effect of additives was tested in 50 mM BICINE, pH 8.0, 150 mM NaCl

Full-length QKI6 appeared mostly as a single band on SDS-PAGE and a major peak in SEC-MALS (Fig. 1a), with an absolute molecular weight of 76 kDa, indicating dimeric state. Some tetramer was present, which might be a disulphide artifact. Mass spectrometry confirmed the correct monomeric mass of QKI6 in reducing conditions (Table 1). Under non-reducing conditions, several masses were observed (Table 1), which decreased to the expected one when TCEP was added. This indicates oxidation sensitivity: the lowest mass could correspond to an intermolecular disulphide bond, as the mass is 1 Da less per monomer. For the remaining peaks, other amino acids need to be considered. QKI6 contains two Cys and 13 Met, and several Met reside in the QUA1 and QUA2 domains (Fig. 1b). The measured mass difference could arise from the oxidation of some Met residues, since the mass increment is 16 Da.

**Table 1:** Mass determination of QKI6 in different redox conditions

Condition	Determined mass (Da)	Δ_mass_ (Da)^a^
0.5 mM TCEP	35,186.5	–
Non-reducing	35,185.5	− 1.0
	35,321.0	+ 134.5
	35,337.0	+ 134.5 + 16
	35,353.0	+ 134.5 + 16 + 16

^a^Δ_mass_ = mass_non-reducing_ − mass_reducing_

DSF was used to screen the impact of salt and buffer on the *T*_m_ of QKI6 (Fig. 1c). QKI6 was most stable in slightly alkaline conditions, peaking at 57–59 °C at pH 8.0. At pH 6.0, melting occurred early, in a heterogeneous manner. The presence of 150–300 mM NaCl further increased *T*_m_. Phosphate changed the unfolding landscape of QKI6 by introducing a second melting event at 71 °C. The effect was reproducible in slightly alkaline conditions and not observed with other buffers (Additional file 1: Fig. S1). Phosphate ions might interact with the RNA-binding cleft of QKI6, stabilizing a population or a region of the protein. (NH_4_)_2_SO_4_, while having a slight stabilizing effect, only presented a single melting event (Fig. 1c).

**Fig. 2:**
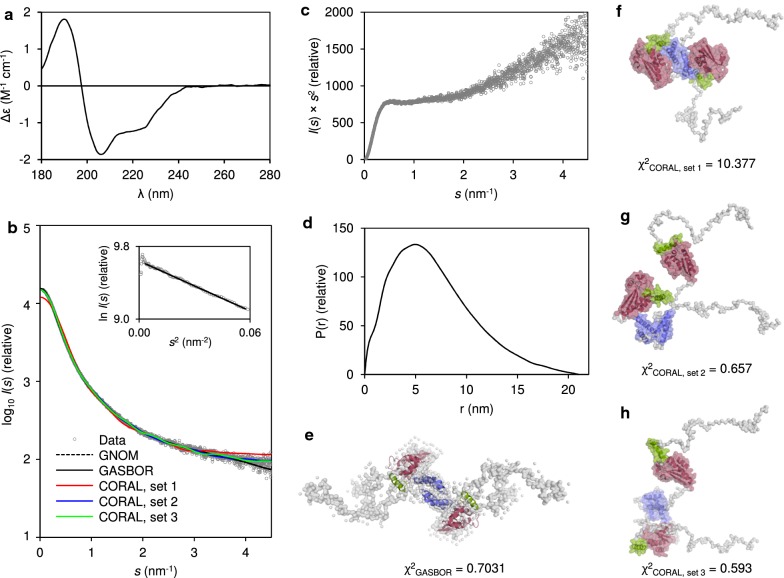
Structure of QKI6. **a** SRCD spectrum of QKI6 reveals significant secondary structure content. **b** SAXS data (open circles) with analysis fits (lines). The inset shows the Guinier region, with a linear fit (solid line). **c** Kratky plot of QKI6 displays high flexibility. **d** Distance distribution diagram from GNOM. **e** GASBOR Ab initio model (gray surface) of QKI6 is elongated. The QUA1, QUA2, and KH subdomain crystal structures have been individually placed inside the model. **f** CORAL model with the fully fixed STAR domain crystal structure (PDB ID: 4jvh [19]) and modelled loops and termini (set 1), **g** fixed QUA1 subdomain dimer with respect to mobile KH-QUA2 subdomains (set 2), and **h** fully separated mobile QUA2 and KH subdomains, with only a fixed QUA1 dimer (set 3). In all CORAL models, the QUA1 dimerization is fixed and based on the crystal structure of QUA1 (PDB ID: 4dnn [16]). In **e**–**h**, all QKI subdomains are colored as in Fig. 1b

Secondary structure prediction of QKI6 suggested that the C-terminal third is unstructured (Fig. 1b). SRCD measurements of QKI6 produced a spectrum typical for a folded protein, but the minimum at 205 nm suggested the presence of disorder (Fig. 2a). This drove us to characterize QKI6 using SAXS (Fig. 2b–h, Additional file 1: Table S1). The dimeric QKI6 was highly flexible, as evident from the Kratky plot (Fig. 2c), and elongated, based on its radius of gyration (*R*_g_, 5.24 nm) and maximum dimension (*D*_max_, 21 nm). Ab initio models based on the SAXS data appear elongated with a compact core (Fig. 2e). Within this core, the individual subdomains of the STAR domain could be fitted in, but only if separated from one another, implying that the crystal structure may not represent the conformation of the STAR domain without bound mRNA.

To verify the flexibility of the STAR domain, we used CORAL to model the missing parts of the protein (Fig. 2f–h). First, we prepared a theoretical dimeric model, where we superposed two STAR domains (PDB ID: 4jvh [19]) on a QUA1 dimer (PDB ID: 4dnn [16]). Then, we performed the analysis in three sets: a dimer of the STAR domain with all subdomains fixed in place (set 1), a fixed dimeric QUA1 domain with linkers to mobile KH-QUA2 units (set 2), and a fixed dimeric QUA1 domain with linkers connecting fully mobile KH and QUA2 subdomains (set 3). The C-terminal region was built as dummy residues. Based on the results, we could clearly exclude set 1 (Fig. 2b, f), χ^2^ being high. In sets 2 and 3, the KH-QUA2 subdomains were clearly separated from the QUA1 dimer, implying a high degree of flexibility. In both sets, the χ^2^ values were much lower (Fig. 2b, g–h). The differences between sets 2 and 3 are marginal, as SAXS cannot distinguish the movement of a single helix (QUA2) with respect to the KH subdomain within the entire protein. Nevertheless, KH and QUA2 are likely to be mobile with respect to each other [18]. This is supported by the fact that the KH-QUA2 unit could not be fitted well within the GASBOR model, but the two subdomains had to be separated (Fig. 2e). In all three modelling sets, the 115 C-terminal residues were extended, in agreement with secondary structure predictions. To conclude, in the absence of an mRNA binding partner, the subdomains of dimeric QKI6 present a great degree of flexibility with respect to each other and most likely collapse to a more ordered arrangement upon binding to a 3′-UTR. The STAR domain is followed by an intrinsically disordered C terminus of currently unknown function.

### Conclusions

We performed a structural characterization of full-length human QKI6. In the absence of RNA binding, the STAR domain is likely to be flexible, with QUA1 separated from KH-QUA2 by a flexible linker. Phosphate changes the thermal unfolding behaviour of QKI6, possibly by interacting with the RNA-binding site. The role of the disordered C terminus is ambiguous, and further studies are required to understand its function in vivo.

## Limitations

The structural characterization employed low-resolution methods, preventing analysis of the fine molecular details of QKI6. Furthermore, as the molecule is flexible, the shown 3D conformations are single snapshots of conformations in the whole ensemble. All experiments here were performed without RNA partners, and it is therefore unclear, exactly how phosphate stabilizes QKI6.

## Supplementary information

**Additional file 1: Table S1.** Small-angle X-ray scattering parameters and analysis. **Fig. S1.** Thermal stability of QKI6 in mildly alkaline conditions. Raw traces of thermal stability experiments (each condition in duplicate) demonstrate the presence of a second melting event at around 71 °C when Na phosphate is used as a buffer/additive. Tris–HCl and BICINE only produced a single melting event over the tested pH range.

## Data Availability

The datasets used and/or analyzed during the current study are available from the corresponding author on reasonable request.

## Abbreviations

OG: oligodendrocyte
MBP: myelin basic protein
QKI: quaking
STAR: signal transduction and activation of RNA
KH: K-homology
TEV: tobacco etch virus
TCEP: tris(2-carboxyethyl)phosphine
NTA: nitrilotriacetic acid
SEC-MALS: size exclusion chromatography-multi-angle light scattering
SRCD: synchrotron radiation circular dichroism
DSF: differential scanning fluorimetry
SAXS: small-angle X-ray scattering
