# Supplementary material for: Stability and flexibility of full-length human oligodendrocytic QKI6
Source: BMC Res Notes. 2019 Sep 23;12:609. doi: 10.1186/s13104-019-4629-x (PMC6757426; doi:10.1186/s13104-019-4629-x)
Supplement: Supplementary file 1 — Additional file 1: Table S1. Small-angle X-ray scattering parameters and analysis. Fig. S1. Thermal stability of QKI6 in mildly alkaline conditions. Raw traces of thermal stability experiments (each condition in duplicate) demonstrate the presence of a second melting event at around 71 °C when Na phosphate is used as a buffer/additive. Tris–HCl and BICINE only produced a single melting event over the tested pH range. [file 13104_2019_4629_MOESM1_ESM.docx]

**Additional Table S1. Small-angle X-ray scattering parameters and analysis.**

| **Data collection parameters** | |
| --- | --- |
| Instrument | P12, PETRAIII, DESY |
| Wavelength (nm) | 0.124 |
| Angular range (nm^-1^) | 0.029 - 4.488 |
| Exposure time per frame (s) | 0.045 |
| Amount of frames | 20 |
| Measurement T (°C) | 10 |
| Concentration range (mg ml^-1^) | 2.3 – 9.0 |
| **Structural parameters** | |
| *I*_0_ (relative) [from Guinier] | 15347.2 |
| *R*_g_ (nm) [from Guinier] | 5.24 |
| *I*_0_ (relative) [from P(r)] | 15760 |
| *R*_g_ (nm) [from P(r) ] | 5.67 |
| D_max_ (nm) [from GNOM] | 21.0 |
| Porod V (nm^3^) [from GNOM] | 167.2 |
| **Molecular mass determination** | |
| Molecular mass M_r_ (kDa) [theoretical dimer] | 70.4 |
| Molecular mass M_r_ (kDa) [from *I*_0_ using Guinier] | 86.0 |
| Molecular mass M_r_ (kDa) [from *I*_0_ using P(r)] | 88.3 |
| Molecular mass M_r_ (kDa) [from Porod V] | 98.4 |
| **Software** | |
| Primary data reduction & processing | BioXTAS RAW, PRIMUS |
| *Ab initio* modeling | GASBOR |
| P2 symmetry, χ^2^ | 0.7031 |
| Modeling of flexibility | CORAL |
| Set 1, χ^2^ | 10.377 |
| Set 2, χ^2^ | 0.657 |
| Set 3, χ^2^ | 0.593 |


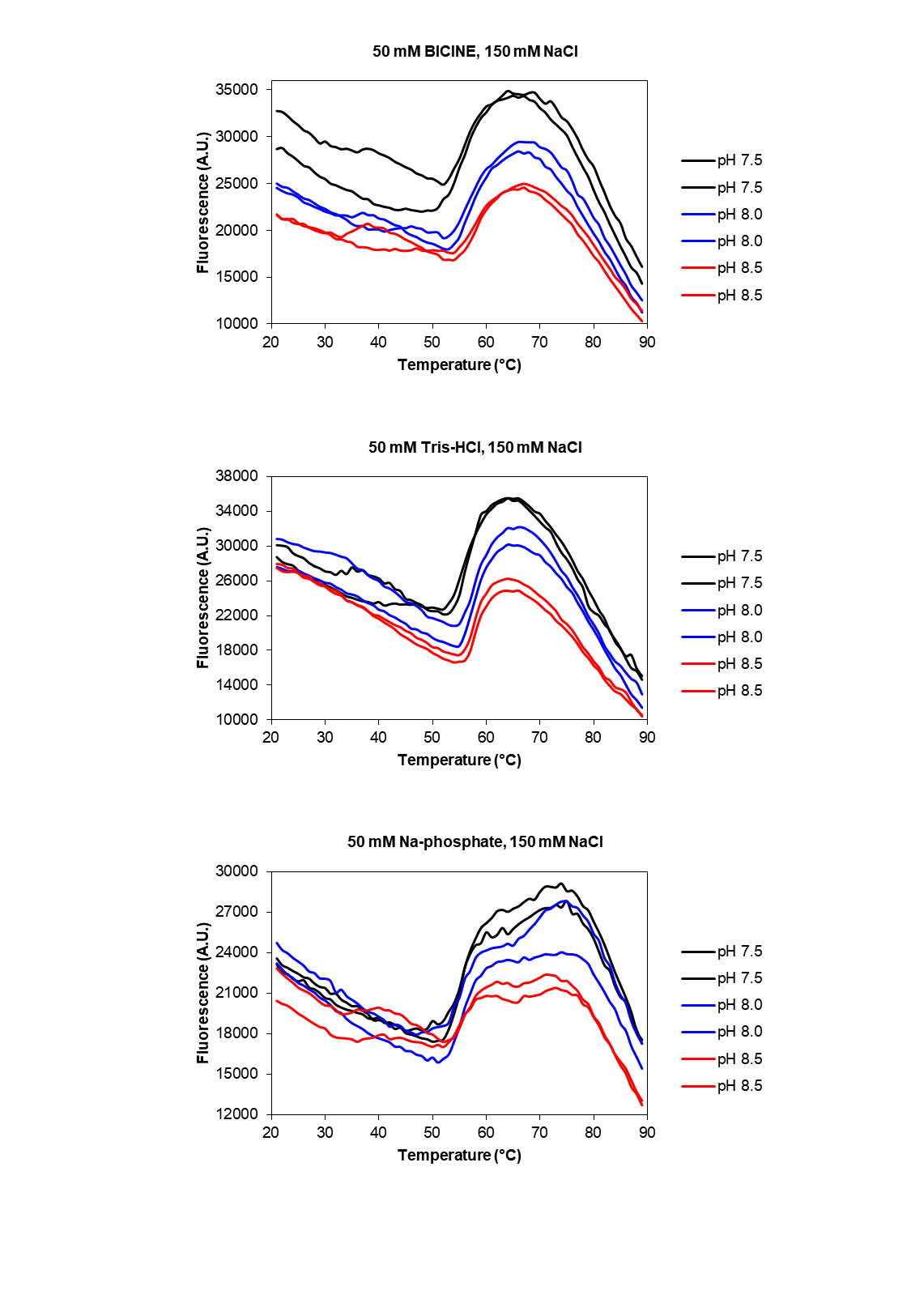


**Additional Fig. S1. Thermal stability of QKI6 in mildly alkaline conditions.** Raw traces of thermal stability experiments (each condition in duplicate) demonstrate the presence of a second melting event at around 71 °C when Na phosphate is used as a buffer/additive. Tris-HCl and BICINE only produced a single melting event over the tested pH range.
